# The impact of education level on all-cause mortality in patients with atrial fibrillation

**DOI:** 10.1038/s41598-024-74478-2

**Published:** 2024-10-25

**Authors:** Áron Sztaniszláv, Anna Björkenheim, Anders Magnuson, Ing-Liss Bryngelsson, Nils Edvardsson, Dritan Poci

**Affiliations:** 1 Department of Cardiology, School of Medical Sciences, Örebro University, Örebro, Sweden; 2 Clinical Epidemiology and Biostatistics, School of Medical Sciences, Faculty of Medicine and Health, Örebro University, Örebro, Sweden; 3 Department of Occupational and Environmental Medicine, Faculty of Medicine and Health, Örebro University, Örebro, Sweden; 4 Sahlgrenska Academy at Sahlgrenska University Hospital, Gothenburg, Sweden; 5 Department of Clinical Physiology, Sahlgrenska University Hospital, Gothenburg, Sweden; 6 Faculty of Medicine and Health, Örebro University, Örebro, Sweden

**Keywords:** Atrial fibrillation, All-cause mortality, Socioeconomic status, Education level, Cardiology, Epidemiology

## Abstract

The association of socioeconomic status with cardiovascular morbidity and mortality is well known, but data on the influence of education level on mortality in individuals with atrial fibrillation (AF) are scarce. We investigated education level as a predictor of all-cause mortality in patients diagnosed with AF. This retrospective cohort study used a database created from several Swedish nationwide registries to identify all patients hospitalized with a diagnosis of AF hospitalized from 1995 to 2008. Education level was categorized as primary, secondary, and academic. All-cause mortality risk was estimated in subpopulations defined by the Charlson Comorbidity Index and several comorbidities. A total of 272,182 patients (56% male; mean age 72 ± 10 years) were followed for five years. Cox regression models showed a reduction in all-cause mortality risk with increased education level. Hazard ratios (HR) relative to primary education remained significant after stratification and adjustment for several confounders: secondary education HR = 0.88; 95% CI: 0.86–0.89; *P* < 0.001; academic education HR = 0.70; 95% CI: 0.67–0.72; *P* < 0.001. Subpopulation analyses confirmed a significant reduction in relative risk with higher education level. Targeted screening and education programs could be effective in reducing mortality in AF patients with fewer years of formal education.

**Supplementary Information:**

The online version contains supplementary material available at 10.1038/s41598-024-74478-2.

## Introduction

Socioeconomic status (SES) significantly influences the incidence, prevalence, and mortality of cardiovascular disease^1,2^. It is a composite measure that encompasses multiple dimensions of social and economic standing, including income, education level, occupation, and factors such as living conditions and geographical deprivation. These variables are often supplementary and inter–dependent and they may provide different perspectives on the same construct^3^. Education level impacts not only an individual’s prospects for income and long-term employment and, by extension, SES, but may reflect cognitive ability, cultural knowledge, and health literacy^4,5^. This quantifiable variable can serve as a tool for directing healthcare resources to targeted prevention, screening, follow-up, and treatment/rehabilitation programs^6,7^.

Atrial fibrillation (AF) is a common arrhythmia, characterized by uncoordinated atrial electrical activation and consequently ineffective atrial contraction^8^. Atrial fibrillation contributes to morbidity and mortality, but the association of AF outcomes with education level is not fully understood.

A systematic review found a correlation between low SES and adverse outcomes in AF, including lower adherence to treatment, poorer prognosis, and increased mortality^9^. The authors did not find a consistent association of SES with risk of developing AF. In contrast, a recent population-wide study showed 12% greater AF incidence in the poorest versus the wealthiest areas of the UK, as measured by the English Indices of Deprivation, which describe local area deprivation, with limited access to education, skill, and training being important domains^6^. Within the Atherosclerosis Risk in Communities (ARIC) study population, individuals with higher income and education levels exhibited a lower incidence of AF up to eighty years of age. Low SES was linked to a decreased lifetime risk of AF, which may be attributed to the longer lifespans typically seen in individuals with higher SES^10^. A Danish nationwide register-based study found that the degree of education inequality in cardiovascular disease varied with diagnosis. The greatest variation was observed in patients with coronary artery disease, acute myocardial infarction, heart failure, and stroke, but no significant difference in education level was observed in AF incidence. The association of education with mortality was not analysed^11^.

The aim of our study was to investigate attained education level as a predictor of all-cause mortality among patients diagnosed with AF.

## Methods

### Data source

The study population has been previously described^12^. Briefly, data from the Swedish National Patient Registry, the General Population Registry, and the Swedish Cause of Death Registry were cross-linked through the Epidemiological Centre of the Swedish National Board of Health and Welfare and Statistics Sweden.

These nationwide registries facilitate the collection of patient data, including age and sex, using personal identity numbers unique to each resident of Sweden. Sweden has a comprehensive public health insurance system, ensuring high-quality data in the registries^13^. All diagnoses cited in this study were based on the International Classification of Diseases (ICD), utilizing the ICD-9 system from 1987 to 1996 and the ICD-10 system from 1997 to the present.

We calculated cumulative Charlson Comorbidity Index (CCI) scores for AF patients to analyse the association of education level with all-cause mortality in the presence of other diseases. The CCI was developed to provide an estimate of mortality risk associated with 20 comorbid conditions, including cardiovascular disease^14,15^. Identification and coding of comorbidities using ICD-9 and ICD-10 was conducted according to the recommendation of Quan et al^16^. In the absence of a universally adopted CCI categorization for cardiac disease^15^, we classified the CCI scores as 0–2 (low mortality risk), 3–4 (moderate mortality risk), and ≥ 5 (high mortality risk). Because of the low number of individuals and deaths, the first two categories (0–4) were merged into a low mortality risk classification, predominantly comprising the original moderate risk category. If multiple diagnostic codes were present within a comorbid condition group, they were counted as one.

We used the CHA_2_DS_2_-VASc score^17^ to describe the thromboembolic risk of the patients.

### Study design and population

This nationwide, register-based, retrospective cohort study encompassed all individuals hospitalized in Sweden with a primary or secondary diagnosis of AF registered for the first time during an index admission from 1 January 1995 through 31 December 2008. Exclusion criteria were unknown education status and age under 30 years to ensure high-quality data on the highest education level. Patients over 85 years of age at diagnosis were not included. (10) Mortality endpoint data were available through 31 December 2009.

Atrial fibrillation was defined as 427 D (DA, DB, DC, DD, and DW) in ICD-9, and I48 and I48.9 (A, B, C, D, E, F, P, and X) in ICD-10. To minimize the risk of information bias, we did not distinguish among paroxysmal, persistent, and permanent AF or atrial flutter (AFl). Our intention was to avoid missing any AF diagnoses, and our choice of study population allowed patients with an AFl diagnosis without concomitant or alternating AF to be included. Validation of AF diagnoses in Danish registries showed that the ICD codes had a high positive predictive value for AF. Specific codes for AFl were rarely used, and the registries were unreliable in distinguishing between the two diagnoses^18^. Allowing patients with both primary and secondary diagnoses of AF meant that our population included both patients in whom AF was the cause of admission and patients in whom AF was detected when they were admitted for other reasons.

The study population was divided into three cohorts based on the highest level of education according to the categories of the International Standard Classification of Education (ISCED) as follows: primary (ISCED 0–2), secondary (ISCED 3–4), and academic (ISCED 5–8) education. After a 30-day blanking period, each subject was followed for up to five years, until death, emigration, or the end of the study on 31 December 2009.

Risk of all-cause mortality was evaluated in subpopulations comprising CCI risk score categories, heart failure, coronary artery disease (CAD), acute myocardial infarction (AMI), and cerebrovascular event (CVE) [stroke, ischaemic stroke, and transient ischaemic attack (TIA)] as well as cancer present at index hospitalization or diagnosed in the preceding five years. The ICD code definitions for stratification factors were based on Andersson et al^12^. To reduce misclassification and accurately assess the impact of CVE, we combined stroke, ischaemic stroke, and TIA into a single variable.

### Statistical analysis

Categorical variables are presented as percentages and continuous variables as mean ± SD. Examined comorbidities were compared using the Chi-squared test and age using one-way analysis of variance (ANOVA) across education groups.

Unadjusted Kaplan-Meier failure plots were used to illustrate the cumulative all-cause mortality risk across education groups, and Cox regression models were used to compare all-cause mortality risk of education groups separately by sex. Cox regression provides hazard ratio (HR) with 95% confidence interval (CI) as association measure. The analysis was conducted unadjusted and adjusted for age, year category of AF diagnosis (1995–1999; 2000–2004; 2005–2008), AF as the primary diagnosis at hospitalization, and CCI score with eight categories (1, 2, 3, 4, 5, 6, 7, ≥ 8). Age was modeled using a restricted cubic spline with four knots according to Harrell^19,20^. Because of non-proportional hazards, stratified Cox regression was used. Non-proportional hazards were evaluated with Schoenfeld residuals and tested with the STATA PHTEST command, with a p value < 0.01 regarded as significant for non-proportionality.

To evaluate whether adjusted mortality risks across education groups differed in patients with low (0–4 score) and high (> 5 score) CCI scores, interaction modelling was performed. A similar interaction analysis was performed to assess mortality risk across education groups for patients with predefined comorbidity diagnoses (heart failure, CAD, AMI, CVE, cancer). To avoid over-adjustment by using the CCI score, the models were adjusted for hypertension and the comorbidities represented by the CCI score (diabetes, peripheral vascular disease, dementia, chronic kidney disease, chronic obstructive pulmonary disease, chronic pulmonary disease, rheumatic disease, mild liver disease, moderate or severe liver disease, hemiplegia or paraplegia, renal disease, HIV, and peptic ulcer), except for CAD and AMI, respectively.

When evaluating the potential interaction of education level with CAD, AMI was not included. In the interaction analysis, non-proportional associations were evaluated by interactions with follow-up time (30 days–2.5 years and > 2.5–5 years follow-up, or 30 days–1 year, > 1–2.5 years, and > 2.5–5 years).

All statistical analyses were performed using STATA versions 16 and 17 (StataCorp, College Station, TX, USA).

### Ethics

The study procedures complied with the principles of the Declaration of Helsinki^21^ and were approved by the Regional Ethical Review Board in Uppsala, Sweden (Dnr 2009/273). All data were anonymised by the Swedish National Board of Health and Welfare and Statistics Sweden prior to being provided to the authors, and requirement for informed consent was waived by the ethical board.

## Results

### Population and descriptive statistics

In total, 272,182 patients received an AF diagnosis at hospitalization during the observation period. We excluded 1,448 patients younger than 30 years and 7,562 for whom education status was not available. A censored event occurred in 13,706 patients during the thirty-day blanking period. Ultimately, 249,466 patients, 44% female, were followed in the study, resulting in 894,737 person-years of observation.

The mean age was 72.2 (10.4) years. Females were significantly older than males, with a mean age of 74.6 (9.0) compared to 70.3 (11.1) years for males (*p* < 0.001). Females accounted for 38% of those in the secondary education category and 33% of those in the academic category. The index hospitalization occurred at a younger age in those of both sexes with higher education. Among patients with primary education only, initial hospitalization with an AF diagnosis occurred at or above age 75 years in 71% of females and 54% of males compared to 37% and 29% of those with academic qualifications, respectively.

Individuals with lower education had a higher stroke risk according to the CHA_2_DS_2_-VASc score. This difference was more pronounced in females of all ages: 53% of those with primary education had a stroke risk score ≥ 4, as opposed to 38% with secondary education and 26% with academic education. For males, the comparable figures were 44%, 31%, and 24%. A similar pattern was observed in the comorbidities assessed by the CCI. High mortality risk was found in 66% of females and 57% of males with primary education, compared to 50% and 43% with secondary education, and 39% and 33% with academic education, respectively (Table 1).

**Table 1:** Descriptive statistics of the study population, stratified by sex; comparison using Chi^2^ test for categorical variables and ANOVA for continuous variables.

Variables	Education Level			
	Primary	Secondary	Academic	Chi^2^*p*
*n* = 249,466	142,134	75,356	31,976	-
**Sex – Female (%)**	70,048 (49.3)	28,527 (37.9)	10,578 (33.1)	-
**Age (Mean ± SD)**	76.7 ± 7.2	71.9 ± 10.2	68.7 ± 11.2	*p* < 0.001†
**Age Categories**,** (n (%))**				
< 65	4,855 (6.9)	6,195 (21.7)	3,373 (31.9)	*p* < 0.001
65–74	16,037 (22.9)	8,411 (29.5)	3,374 (31.9)	
75–85	49,516 (70.2)	13,921 (48.8)	3.831 (36.2)	
**Stroke risk – CHA**_**2**_ **DS**_**2**_ -VASc score categories* (n (%))				
0 – Low risk	2,603 (3.7)	3,774 (13.2)	2,388 (22.6)	*p* < 0.001
1 – Moderate risk	7,668 (11.0)	4,912 (17.2)	2,345 (22.2)	
2 – OAC indication risk	5,248 (7.5)	3,097 (10.9)	1,173 (11.1)	
3 – High risk	17,177 (24.5)	5,892 (20.1)	1,926 (18.2)	
≥ 4 – Very high risk	37,352 (53.3)	10,852 (38.0)	2,746 (26.0)	
**Comorbidities – CCI score categories (n (%))**				
Low mortality risk	1,546 (2.2)	2,700 (9.5)	1,741 (16.6)	*p* < 0.001
Moderate mortality risk	22,060 (31.5)	11,372 (40.0)	4,740 (44.8)	
High mortality risk	46,442 (66.3)	14,455 (50.1)	4,097 (38.7)	
**Sex – Male n (%)**	72,086 (50.7)	46,829 (62.1)	21,398 (66.9)	-
**Age (Mean ± SD)**	73.1 ± 9.3	68.0 ± 12.0	65.9 ± 12.1	*p* < 0.001†
**Age Categories (n (%))**				
< 65	12,524 (17.4)	16,226 (34.7)	8,943 (41.8)	*p* < 0.001
65–74	21,613 (30.0)	13,713 (29.3)	6,469 (30.2)	
75–85	37,949 (52.6)	16,890 (36.1)	5,986 (28.0)	
**Stroke risk – CHA**_**2**_ **DS**_**2**_ -VASc score categories* (n (%))				
0 – Low risk	5,985 (8.3)	9,118 (19.5)	5,744 (26.8)	*p* < 0.001
1– Moderate risk	10,517 (14.6)	8,764 (18.7)	4,420 (20.7)	
2 – OAC indication risk	8,451 (11.7)	6,019 (12.9)	2,758 (12.9)	
3 – High risk	15,215 (21.1)	8,278 (17.7)	3,388 (15.8)	
≥ 4 – Very high risk	31,918 (44.3)	14.650 (31.3)	5,088 (23.8)	
**Comorbidities – CCI score categories (n (%)**				
Low mortality risk	4,765(6.6)	8,459 (18.1)	5,084 (23.8)	*p* < 0.001
Moderate mortality risk	26,099 (36.2)	18,130 (38.7)	9,193 (43.0)	
High mortality risk	41,222 (57.2)	20,240 (43.2)	7,121 (33.3)	

CCI , Charlson Comorbidity Index; OAC, Oral anticoagulant.

CCI score 0–2 = low mortality risk; CCI score 3–4 = moderate mortality risk; CCI score ≥ 5 = high mortality risk.

† One way ANOVA analysis *p* < 0.001.

*For the CHA_2_DS_2_-VASc score, sex was not considered a risk factor as the analysis was performed stratified by sex.

Significant differences were found in the incidence of all assessed comorbidities across education levels, with patients having academic education showing a lower comorbidity burden (Table 2).

**Table 2:** Descriptive statistics of prognostic factors in the population, stratified by sex; comparison using Chi^2^ test for categorical variables and ANOVA for continuous variables.

Variables	Education Level			
	Primary	Secondary	Academic	Chi^2^*p*
*n* = 249,466	142,134	75,356	31,976	-
**Sex – Female n (%)**	70,048 (49.3)	28,527 (37.9)	10,578 (33.1)	-
**Female**				
Hypertension n (%)	29,957 (28.5)	8,156 (28.6)	2,536 (24.0)	*p* < 0.001
Diabetes n (%)	9,804 (14.0)	3,095 (10.9)	619 (6.5)	*p* < 0.001
Acute myocardial infarction n (%)	7,639 (10.9)	2,621 (9.2)	684 (6.5)	*p* < 0.001
Heart failure n (%)	18,662 (26.6)	5,831 (20.4)	1,530 (14.5)	*p* < 0.001
Coronary artery disease n (%)	17,218 (24.6)	5,974 (20.9)	1,573 (14.9)	*p* < 0.001
Cerebrovascular event n (%)	11,344 (16.2)	3,834 (13.4)	1,168 (11.0)	*p* < 0.001
Cancer n (%)	12,256 (17.5)	5,546 (19.4)	2,104 (19.9)	*p* < 0.001
Chronic kidney disease n (%)	798 (1.1)	298 (1.0)	89 (0.8)	*p* = 0.02
COPD n (%)	3,325 (4.8)	1,432 (5.0)	311 (2.9)	*p* < 0.001
**Sex – Male n (%)**	72,086 (50.7)	46,829 (62.1)	21,398 (66.9)	-
Hypertension n (%)	17,266 (24.0)	11,465 (24.5)	4,903 (22.9)	*p* < 0.001
Diabetes n (%)	10,983 (15.2)	6,021 (12.9)	2,177 (10.2)	*p* < 0.001
Acute myocardial infarction n (%)	10,820 (15.0)	6,018 (12.9)	2,166 (10.1)	*p* < 0.001
Heart failure n (%)	20,415 (28.3)	10,583 (22.6)	3,604 (16.8)	*p* < 0.001
Coronary artery disease n (%)	22,771 (31.6)	12,809 (27.4)	4,681 (21.9)	*p* < 0.001
Cerebrovascular event n (%)	10,757 (14.9)	5,715 (12.2)	2,338 (10.9)	*p* < 0.001
Cancer n (%)	10,946 (15.2)	6,493 (13.9)	2,647 (12.4)	*p* < 0.001
Chronic kidney disease n (%)	1,370 (1.9)	896 (1.9)	299 (1.4)	*p* < 0.001
COPD n (%)	4,060 (5.6)	2,078 (4.4)	513(2.4)	*p* < 0.001

COPD, Chronic obstructive pulmonary disease.

Cerebrovascular event was defined as a composite of the history of ischemic stroke, transient ischemic attack, and stroke without further specification. Each type of event was counted only once per patient.

### Education and all-cause mortality

**Figure 1:**
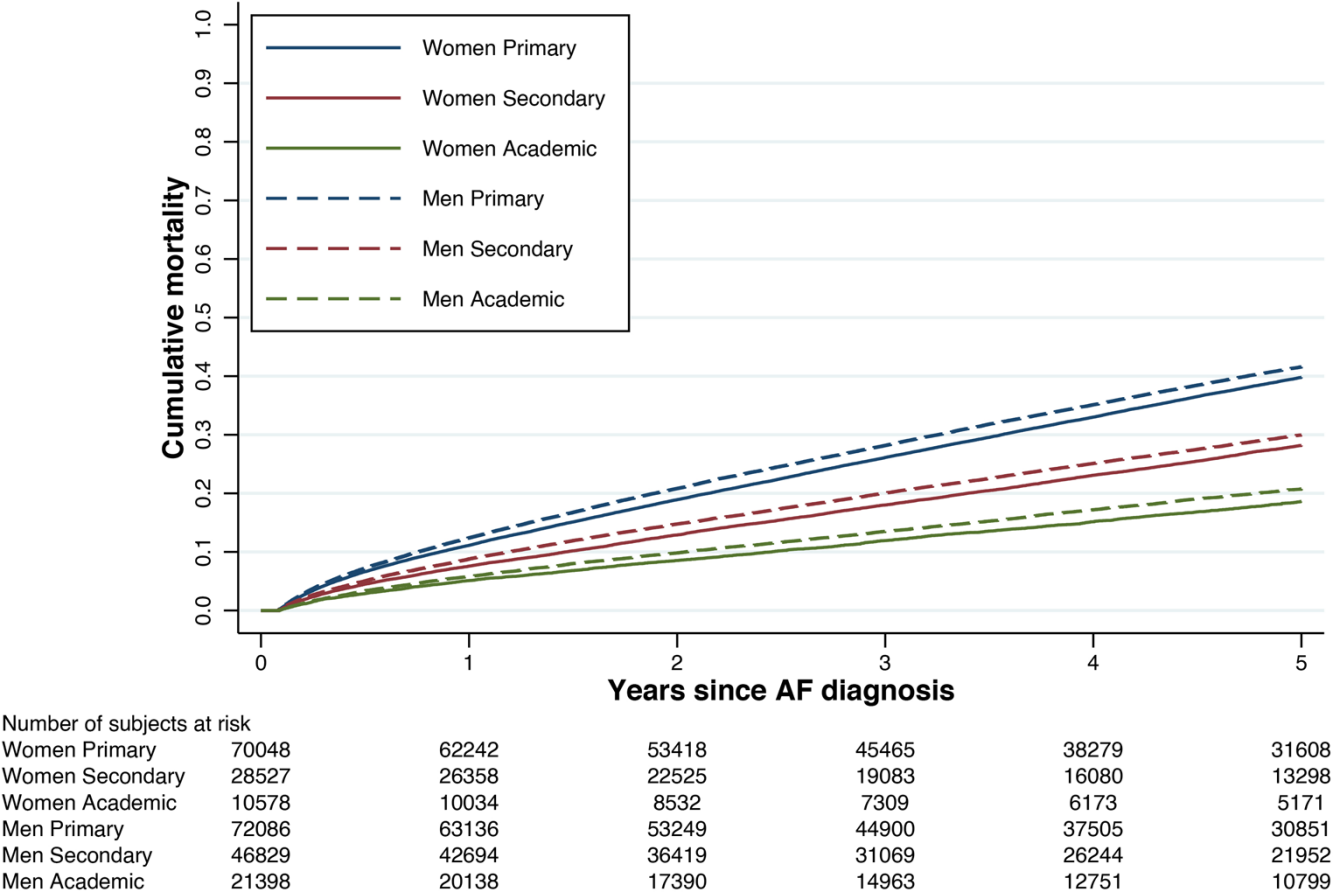
Kaplan-Meier failure estimates by education level, stratified by sex.

The unadjusted Kaplan-Meier plots showed the highest rate of all-cause mortality to be in patients with only primary education. Across education levels, all-cause mortality was lower in females than in males (Fig. 1).

The unadjusted and adjusted Cox regression analyses revealed a significant association between education level and all-cause mortality in both sexes.

In females, the adjusted HR was 0.87 (95% CI: 0.84–0.89) when comparing secondary to primary education, and 0.71 (95% CI: 0.67–0.74) when comparing academic to primary education. In males, the adjusted HR was 0.89 (95% CI: 0.87–0.91) when comparing secondary to primary education, and 0.72 (95% CI: 0.69–0.74) when comparing academic to primary education (Table 3).

**Table 3:** Comparison of five-year all-cause mortality risk across education levels using cox regression, stratified by sex.

Female (*n* = 109,153)	*N*	Events (%)	Rates	Unadjusted^1^ HR (95% CI)	Adjusted^2^ HR (95% CI)
Education level					
Primary	70,048	25,493 (36.4)	102.2	Ref	Ref
Secondary	28,527	6,981 (24.5)	66.7	0.65 (0.64–0.67)	0.87 (0.84–0.89)
Academic	10,578	1,665 (15.7)	41.8	0.41 (0.39–0.43)	0.71 (0.67–0.74)

Male (*n* = 140,313)	N	Events (%)	Rates		Adjusted ^1^ HR (95% CI)
Education level					
Primary	72,086	27,338 (37.9)	109.7	Ref	Ref
Secondary	46,829	12,420 (26.5)	72.9	0.67 (0,65–0.68)	0.89 (0.87–0.91)
Academic	21,398	3,849 (18.0)	47.5	0.44 (0.42–0.45)	0.72 (0.69–0.74)

Crude rates per 1,000 person-years; CI, Confidence interval; HR, Hazard ratio.

^1^Non-proportional hazards were observed in the unadjusted regression models.

^2^Adjusted for age as spline, year of AF diagnosis (1995–99, 2000–04, 2005–08), and AF as primary diagnosis with stratified Cox regression because of non-proportional hazard and CCI score (1–7, ≥ 8).

### Education and all-cause mortality in comorbidity subpopulation

#### Education and CCI score

In the interaction by CCI score analysis, a time-dependent association between education level and all-cause mortality was observed (Table 4). During the initial 2.5 years of follow-up, the association of education with mortality was significantly more pronounced in the low-risk CCI group compared to the high-risk group. The interaction HR was 1.36 (95% CI: 1.24–1.49) for females with secondary education and 1.72 (95% CI: 1.47–2.02) for those with academic education, and 1.22 (95% CI: 1.14–1.30) for males with secondary and 1.43 (95% CI: 1.29–1.58) for males with academic education. At > 2.5 to 5 years, the effect of CCI score on the association was reduced in females: interaction HR 1.02 (95% CI: 0.93–1.13) with secondary education and 1.35 (95% CI: 1.12–1.62) with academic education. In males, the interaction HR was significant: HR 1.12 (95% CI: 1.04–1.21) for secondary education and 1.46 (95% CI: 1.29–1.64) for academic education.

**Table 4:** Comparison of five-year all-cause mortality risks across education levels, stratified by CCI score using cox regression.

*Female (n = 109*,*153)*	*Events*	*Rates*	*Adjusted* ^*1*^ *HR (95% CI)*	
			*Stratified*	*Interactions*
Education level by CCI score				
CCI score 0–4, 30d–2.5 yrs follow-up				
Primary	2,243	41.1	Ref	
Secondary	750	22.9	0.66 (0.61–0.72)	
Academic	208	13.8	0.45 (0.39–0.52)	
CCI score ≥ 5, 30d–2.5 yrs follow-up				
Primary	13,176	139.4	Ref	Ref
Secondary	3,503	117.7	0.90 (0.87–0.94)	1.36 (1.24–1.49)
Academic	824	96.5	0.78 (0.73–0.84)	1.72 (1.47–2.02)
CCI score 0–4, > 2.5–5 yrs follow–up				
Primary	1,914	44.4	Ref	
Secondary	707	28.8	0.86 (0.79–0.94)	
Academic	173	15.6	0.55 (0.47–0.65)	
CCI score ≥ 5, >2.5–5 yrs follow-up				
Primary	8,160	142.8	Ref	Ref
Secondary	2,021	114.6	0.88 (0.84–0.93)	1.02 (0.93–1.13)
Academic	460	90.3	0.74 (0.68–0.82)	1.35 (1.12–1.62)

*Male (n = 140*,*313)*	*Events*	*Rates*	*Adjusted* ^*1*^ *HR (95% CI)*	
			*Stratified*	*Interactions*
Education level by CCI score				
CCI score 0–4, 30d–2.5 yrs follow-up				
Primary	3,381	48.0	Ref	
Secondary	1,823	29.6	0.76 (0.72–0.81)	
Academic	641	19.3	0.54 (0.49–0.59)	
CCI score ≥ 5, 30d–2.5 yrs follow-up				
Primary	13,935	173.6	Ref	Ref
Secondary	6,064	151.5	0.93 (0.90–0.96)	1.22 (1.14–1.30)
Academic	1,759	121.2	0.77 (0.73–0.81)	1.43 (1.29–1.58)
CCI score 0–4, > 2.5–5 yrs follow-up				
Primary	2,662	49.7	Ref	
Secondary	1,424	30.8	0.80 (0.75–0.86)	
Academic	479	19.2	0.55 (0.49–0.60)	
CCI score ≥ 5, >2.5–5 yrs follow-up				
Primary	7,360	163.7	Ref	Ref
Secondary	3,109	137.2	0.90 (0.87–0.94)	1.12 (1.04–1.21)
Academic	970	116.0	0.79 (0.74–0.85)	1.46 (1.29–1.64)

Crude rates per 1000 person-years.

CCI, Charlson Comorbidity Index; CI, Confidence interval; HR, Hazard ratio.

^1^ Adjusted for age as spline, year of AF diagnosis (1995–99, 2000–04, 2005–08), and AF as primary diagnosis with stratified Cox regression and CCI score (1–7, ≥ 8) by follow-up time (30 days–2.5 years, > 2.5–5 years) interactions because of non-proportional hazards.

#### Education and selected comorbidities

**Figure 2:**
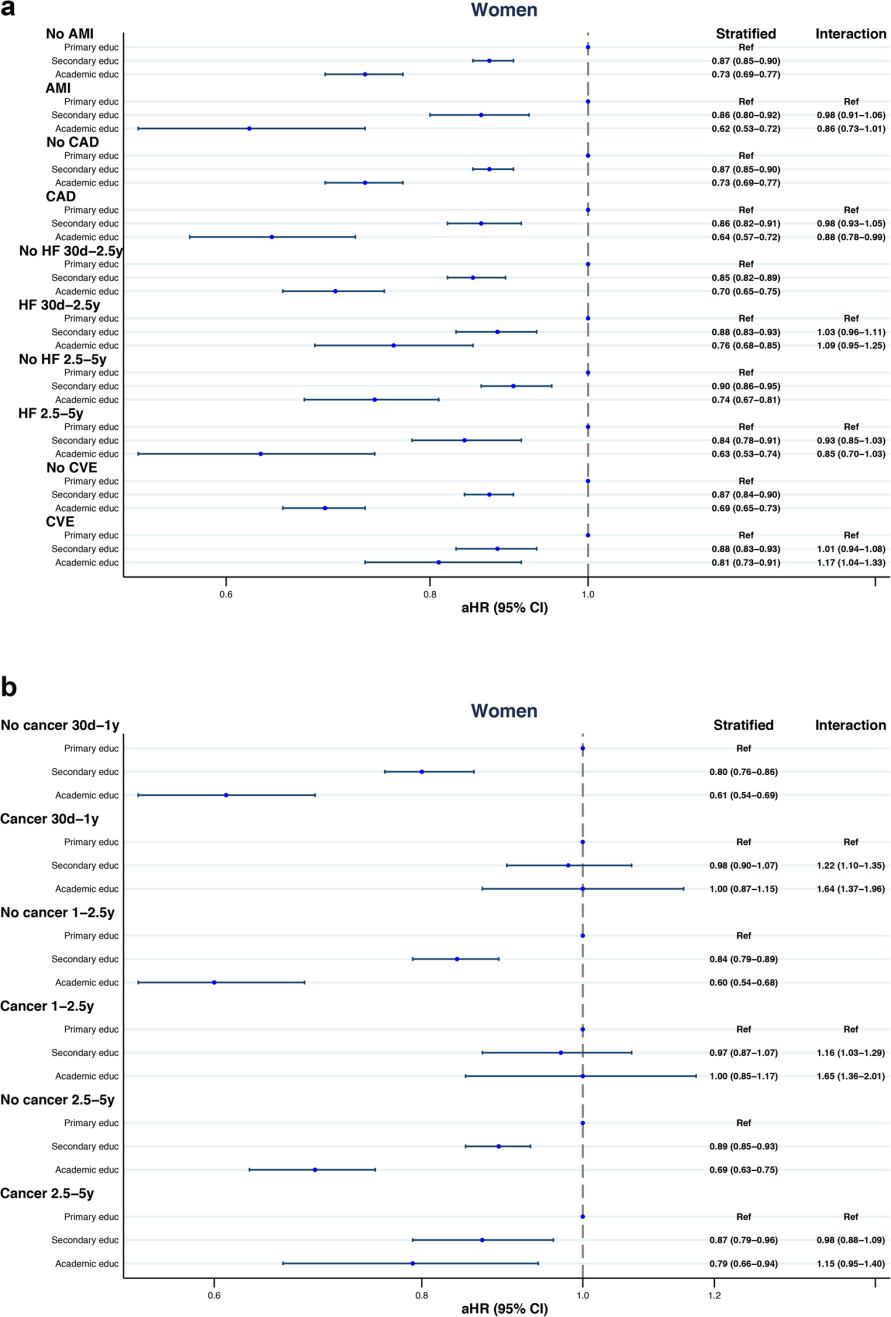
(**a**) Forest plot of five-year all-cause mortality risk across education levels, stratified by selected cardiovascular comorbidities in females. (**b**) Forest plot of five-year all-cause mortality risk across education levels, stratified by cancer in females.

**Figure 3:**
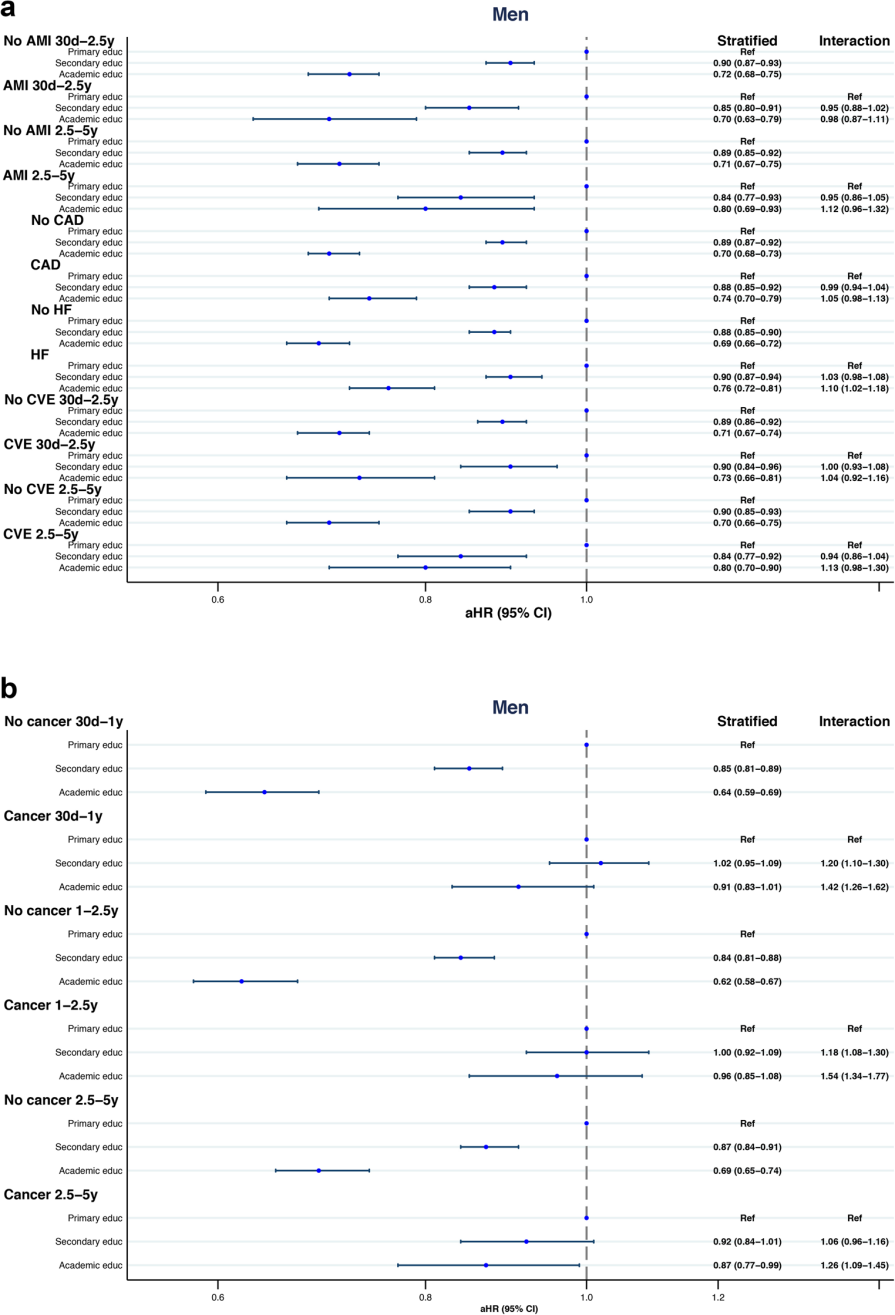
(**a**) Forest plot of five-year all-cause mortality risk across education levels, stratified by selected cardiovascular comorbidities in males. (**b**) Forest plot of five-year all-cause mortality risk across education levels, stratified by cancer in males.

We assessed AMI, CAD, heart failure (HF), CVE, and cancer recorded during the index hospitalization or within the preceding five years to determine their potential impact on the association of education level with all-cause mortality in AF (Fig. 2a-b and 3a-b.; Supplementary Tables 1 and 2).

The association between mortality risk and education level did not significantly interact with AMI or CAD.

Heart failure did not show interaction with education on mortality risk in females, while in males a significant interaction was found. In males, the mortality risk for academic compared to primary education was HR 0.69 (95% CI: 0.66–0.72) in patients without HF and 0.76 (95% CI: 0.72–0.81) in HF patients, with an interaction HR of 1.10 (95% CI: 1.02–1.18).

In females with a history of CVE, all-cause mortality risk was lower in those with academic compared to primary education [HR 0.81 (95% CI: 0.73–0.91)]. In females with no history of CVE, the HR was 0.69 (95% CI: 0.65–0.73), showing an interaction HR of 1.17 (95% CI: 1.04–1.33). This interaction was not observed in males.

With respect to cancer, a time-dependent association was observed between education level and all-cause mortality risk. The model was analysed across three time periods (30 days–1 year; >1–2.5 years; >2.5–5 years) in both males and females. During the first two periods, education level was not significantly associated with mortality. At > 2.5–5 years, both males and females with academic education showed a significantly lower mortality risk compared to those with primary education.

## Discussion

The risk of all-cause mortality during a five-year follow-up of hospitalized patients with AF was lower in those with higher education levels relative to those with only primary education. This positive effect increased with education level and persisted after adjusting for multiple confounders.

Our findings are consistent with those of Patsiou et al., who found significantly lower all-cause mortality among highly-educated AF patients in a retrospective cohort study of 1,082 patients after a median 31-month follow-up^22^. Similarly, in the Cardiovascular Disease in Norway 2008–2012 project, Akerkar et al. prospectively followed 42,138 patients discharged after hospitalization with AF. They reported higher all-cause and cardiovascular mortality in groups with lower education levels over a mean follow-up of 2.4 years^23^. A Swedish cohort study examining 12,283 AF patients from 75 primary care centres, primarily in the Stockholm region, revealed a significantly lower relative mortality risk among patients with higher education levels after adjustment for several socioeconomic factors. Academic education was associated with a lower risk of heart failure in females and a lower risk of AMI in both sexes^24^.

While our study population included individuals diagnosed with AFl as well as AF, we believe that the proportion of patients with AFl (2.5%) was too small to affect overall results.

Our sex-stratified analysis showed comparable mortality rates in males and females, despite higher mean age and greater stroke risk in females at index hospitalization. A systematic review has reported similar results in studies with shorter follow-up periods^25^.

Education level was associated with all-cause mortality in individuals with AF, independent of the primary hospitalization diagnosis. Lower education potentially contributes to a worse prognosis influenced by factors such as low health literacy, limited disease awareness, decreased quality of life^26,27^, and less accessibility to advanced AF treatments such as catheter ablation^9,7^. Poor adherence to prescribed medications is more common in patients with lower education levels and may play a substantial role in adverse outcomes^28^.

Subpopulation analysis stratified by CCI score revealed a positive effect of education in all CCI subgroups. However, the effect on mortality was more pronounced in the low-risk CCI subgroup compared to the high-risk group.

While significant interaction HRs emerged when comparing low/moderate with high comorbidity subgroups, those with higher education still showed reduced mortality risk. The positive effect of education was lower in the early follow-up periods but greater in the long term. This phenomenon might underline the role of health literacy and, consequently, better compliance with treatment. The comorbidities of the lower-risk CCI subgroup, can be largely managed by long-term medication or rehabilitation, which might not be possible in the case of more serious comorbidities. In high-risk CCI subgroups, greater access to advanced therapies can have an additive effect, leading to a relevant difference in all-cause mortality risk.

Notably, neither CAD nor AMI significantly altered the association of education level with all-cause mortality. Heart failure exerted a significant impact on all-cause mortality risk in academically educated males compared to those with primary education. The interaction analysis of CVE showed a significant HR only in academically educated females.

The interaction of cancer analysis showed a time-dependent association. In the initial 2.5 years, the mortality risk in cancer patients was significantly greater, and mortality rates did not differ with education level. In patients surviving beyond 2.5 years, a positive effect of education was evident, although to a lesser extent than observed in patients without cancer. A time-series study from the US examining the data of 8.2 million individuals who died from cancer showed a pronounced education gap in mortality, regardless of the type of malignancy. This effect persisted even after policy changes that made screening programs and treatment more accessible to broader populations^29^. A study from Colombia reported consistent educational disparities in mortality trends even after the implementation of a more general health insurance system^30^. These findings suggest that worldwide data might be comparable, despite differences in healthcare systems.

Our results are in accordance with those of a recent Chinese prospective observation study following stroke patients for mortality and cardiovascular events for two years. This study showed a similar mortality reduction at higher education levels. The relative risk differences were higher in the Chinese population. A meta-analysis showed that comparable studies from Western populations demonstrated a similar protective effect of education, albeit with shorter follow-up periods and smaller populations^31^.

The observed differences may be related to available therapies and the types of morbidity. A lower education level is associated with higher mortality and rehospitalisation risk in cases of CAD, acute coronary syndrome^32^, and heart failure^33^. Our study included patients from the era of thrombolysis as well as the early days of percutaneous coronary intervention, which provides better prognosis for long-term survival. Thus, CAD and AMI did not affect the baseline association between education level and mortality risk. Heart failure requires a high level of compliance with medication and lifestyle changes, factors in which education level may play a prominent role. The time-dependent interaction effect of cancer can be explained similarly. Patients who died within 2.5 years of index hospitalisation may have had advanced disease with a negative prognosis and seen little or no effect of available therapies on the mortality risk. Higher education level and health literacy could at the same time account for early diagnosis and successful treatment.

Observational studies carry a potential for bias that must be considered when interpreting results. Despite utilizing data extracted from high-quality national registries, there is a risk of misclassification of diagnoses and coding errors. In clinical practice, ECG interpretation may not be straightforward, and atypical AFl may be classified as AF and vice versa. AF and AFl can occur simultaneously or alternate in the same patient. Our intention was to not miss any AF diagnoses, and our choice of study population included patients with an AFl diagnosis without known concomitant or alternating AF, accounting for 2.5% of all diagnoses.

As our study cohort was drawn from hospitalized patients, it is reasonable to assume that it may have experienced higher comorbidity and mortality rates compared to a general AF population. Data from primary care were not available but could have helped in determining whether patients who appeared to have incident AF might have had an AF diagnosis before hospital admission.

Although it included cancer and known cardiovascular comorbidity risk factors for mortality in patients with AF, our sub-analyses of the interaction between comorbidities and education level should be considered exploratory^34^.

Even though our data shows a significant association between education attainment and all–cause mortality, several other aspects of SES such as income, might have a potential independent influence on mortality. A national register-based study from Finland examined the association between personal maximal income and the risk of mortality and a first stroke during a 10-year follow-up period. Income showed a significant association with mortality and stroke. The most prominent association was observed in patients under 65 years of age, which is the usual age of retirement in Finland. Education had a significant protection effect only in patients > 75 years, which is close to the mean age of our population^35^. This way this registry is not contradictory to the results of our study.

The strength of this study is the large number of patients and the five-year follow-up based on data from high-quality national registries.

## Conclusions

Targeted screening and education programs may be effective in reducing mortality rates in patients with AF and low education attainment. Several healthcare policies and interventions should integrate educational support in order to improve patient outcomes.

## Supplementary Information

Supplementary Material 1.

Supplementary Material 2.

## Data Availability

The datasets generated during and/or analysed during the current study are available from the corresponding author on reasonable request.
