# Supplementary material for: The impact of education level on all-cause mortality in patients with atrial fibrillation
Source: Sci Rep. 2024 Oct 25;14:25386. doi: 10.1038/s41598-024-74478-2 (PMC11511939; doi:10.1038/s41598-024-74478-2)
Supplement: Supplementary file 1 — Supplementary Material 1. [file 41598_2024_74478_MOESM1_ESM.docx]

***Supplementary Table 1.*** Five-year all-cause mortality risk across education levels, stratified by specific comorbidities, using Cox regression in females

| *Female (n=109,153)* | *Events* | *Crude rates* | *Adjusted ^1^*  *HR (95% CI)* | |
| --- | --- | --- | --- | --- |
|  |  |  | ***Stratified*** | ***Interactions*** |
| Education level by |  |  |  |  |
| Acute myocardial infarction ^2^ |  |  |  |  |
| No |  |  |  |  |
| Primary | 21,880 | 96.9 | Ref |  |
| Secondary | 6,064 | 63.0 | 0.87 (0.85–0.90) |  |
| Academic | 1,499 | 39.9 | 0.73 (0.69–0.77) |  |
| Yes |  |  |  |  |
| Primary | 3,613 | 152.2 | Ref | Ref |
| Secondary | 917 | 107.8 | 0.86 (0.80–0.92) | 0.98 (0.91–1.06) |
| Academic | 166 | 72.1 | 0.62 (0.53–0.73) | 0.86 (0.73–1.01) |
| Coronary artery disease ^2^ |  |  |  |  |
| No |  |  |  |  |
| Primary | 18,295 | 95.3 | Ref |  |
| Secondary | 5,103 | 60.7 | 0.87 (0.85–0.90) |  |
| Academic | 1,334 | 38.9 | 0.73 (0.69–0.77) |  |
| Yes |  |  |  |  |
| Primary | 7,198 | 125.3 | Ref | Ref |
| Secondary | 1,878 | 91.0 | 0.86 (0.82–0.91) | 0.98 (0.93–1.05) |
| Academic | 331 | 59.5 | 0.64 (0.57–0.72) | 0.88 (0.78–0.99) |
| Heart failure |  |  |  |  |
| No 30 days–2.5 years follow-up |  |  |  |  |
| Primary | 9,273 | 82.6 | Ref |  |
| Secondary | 2,715 | 53.6 | 0.85 (0.82–0.89) |  |
| Academic | 717 | 35.1 | 0.70 (0.65–0.75) |  |
| Yes 30 days – 2.5 years follow-up |  |  |  |  |
| Primary | 6,146 | 166.8 | Ref | Ref |
| Secondary | 1,538 | 129.3 | 0.88 (0.83–0.93) | 1.03 (0.96–1.11) |
| Academic | 315 | 98.3 | 0.76 (0.68–0.85) | 1.09 (0.95–1.25) |
| No >2.5–5 years follow-up |  |  |  |  |
| Primary | 6,654 | 85.0 | Ref |  |
| Secondary | 1,910 | 54.8 | 0.90 (0.86–0.95) |  |
| Academic | 489 | 34.6 | 0.74 (0.67–0.81) |  |
| Yes >2.5–5 years follow-up |  |  |  |  |
| Primary | 3,420 | 155.5 | Ref | Ref |
| Secondary | 818 | 111.7 | 0.84 (0.78–0.91) | 0.93 (0.85–1.08) |
| Academic | 144 | 70.4 | 0.63 (0.53–0.74) | 0.85 (0.70–1.03) |
| Cerebrovascular event |  |  |  |  |
| No |  |  |  |  |
| Primary | 20,314 | 95.6 | Ref |  |
| Secondary | 5,657 | 61.6 | 0.87 (0.84–0.90) |  |
| Academic | 1,337 | 37.2 | 0.69 (0.65–0.73) |  |
| Yes |  |  |  |  |
| Primary | 5,179 | 140.1 | Ref | Ref |
| Secondary | 1,324 | 103.5 | 0.88 (0.83–0.93) | 1.01 (0.94–1.08) |
| Academic | 328 | 84.1 | 0.81 (0.73–0.91) | 1.17 (1.04–1.33) |
| Cancer |  |  |  |  |
| No 30 days–1 year follow-up |  |  |  |  |
| Primary | 5,543 | 101.1 | Ref |  |
| Secondary | 1,375 | 61.8 | 0.80 (0.76–0.86) |  |
| Academic | 297 | 35.7 | 0.61 (0.54–0.69) |  |
| Yes 30 days–1 year follow-up |  |  |  |  |
| Primary | 2,249 | 204.9 | Ref | Ref |
| Secondary | 788 | 154.4 | 0.98 (0.90–1.07) | 1.22 (1.10–1.35) |
| Academic | 244 | 123.8 | 1.00 (0.87–1.15) | 1.64 (1.37–1.96) |
| No 1–2.5 years follow-up |  |  |  |  |
| Primary | 6,009 | 85.2 | Ref |  |
| Secondary | 1,543 | 53.0 | 0.84 (0.79–0.89) |  |
| Academic | 320 | 29.1 | 0.60 (0.54–0.68) |  |
| Yes 1–2.5 years follow-up |  |  |  |  |
| Primary | 1,618 | 126.3 | Ref | Ref |
| Secondary | 547 | 90.3 | 0.97 (0.87–1.07) | 1.16 (1.03–1.29) |
| Academic | 171 | 72.2 | 1.00 (0.85–1.17) | 1.65 (1.36–2.01) |
| No 2.5–5 years follow-up |  |  |  |  |
| Primary | 8,297 | 96.2 | Ref |  |
| Secondary | 2,191 | 61.7 | 0.89 (0.85–0.93) |  |
| Academic | 494 | 36.3 | 0.69 (0.63–0.75) |  |
| Yes 2.5–5 years follow-up |  |  |  |  |
| Primary | 1,777 | 126.7 | Ref | Ref |
| Secondary | 537 | 80.8 | 0.87 (0.79–0.96) | 0.98 (0.88–1.09) |
| Academic | 139 | 54.1 | 0.79 (0.66–0.94) | 1.15 (0.95–1.40) |
| ^1^ Adjusted for age as spline, year of AF diagnosis (1995–99, 2000–04, 2005–08) and AF as main admission diagnosis with stratified Cox regression due to non-proportional hazards and the potential comorbidity variables in the table as well as hypertension, diabetes, peripheral vascular disease, dementia, chronic pulmonary disease, rheumatic disease, mild liver disease, moderate to severe liver disease, hemiplegia/paraplegia, renal disease, HIV, and peptic ulcer. Co-morbidities showing non-proportional hazards was evaluated by follow-up time (30 days–2.5 years, >2.5–5 years or 30 days–1 year,> 1–2.5 years, >2.5–5 years) interactions.  ^2^ Not adjusted for CAD due to overlap with AMI and vice versa.  *Significant interaction HR value  HR = Hazard ratio; CI = Confidence interval; crude rate per 1000 person-years.  The cardiovascular event variable is a composite of history of ischemic stroke, transient ischemic attack, and stroke without further specification. Each of these events was counted only once per patient. | | | | |
