# Supplementary material for: The impact of education level on all-cause mortality in patients with atrial fibrillation
Source: Sci Rep. 2024 Oct 25;14:25386. doi: 10.1038/s41598-024-74478-2 (PMC11511939; doi:10.1038/s41598-024-74478-2)
Supplement: Supplementary file 2 — Supplementary Material 2. [file 41598_2024_74478_MOESM2_ESM.docx]

***Supplementary table 2.*** Five-year all-cause mortality risk across education levels, stratified by specified comorbidities, using Cox regression in males

| Males (n=140,313) | Events | Crude rates | Adjusted^1^  HR (95% CI) | |
| --- | --- | --- | --- | --- |
|  |  |  | Stratified | Interactions |
| Education level by |  |  |  |  |
| Acute myocardial infarction ^2^ |  |  |  |  |
| No 30 days–2.5 years follow-up |  |  |  |  |
| Primary | 14,050 | 108.6 | Ref |  |
| Secondary | 6,488 | 72.7 | 0.90 (0.87–0.93) |  |
| Academic | 2,030 | 47.0 | 0.72 (0.68–0.75) |  |
| Yes 30 days–2.5 years follow-up |  |  |  |  |
| Primary | 3,266 | 153.1 | Ref | Ref |
| Secondary | 1,399 | 113.1 | 0.85 (0.80–0.91) | 0.95 (0.88–1.02) |
| Academic | 370 | 80.6 | 0.70 (0.63–0.79) | 0.98 (0.87–1.11) |
| No >2.5–5 years follow-up |  |  |  |  |
| Primary | 8,477 | 98.5 | Ref |  |
| Secondary | 3,858 | 62.9 | 0.89 (0.85–0.92) |  |
| Academic | 1,250 | 40.9 | 0.71 (0.67–0.75) |  |
| Yes 2.5–5 years follow-up |  |  |  |  |
| Primary | 1,545 | 124.1 | Ref | Ref |
| Secondary | 675 | 90.3 | 0.84 (0.77–0.93) | 0.95 (0.86–1.05) |
| Academic | 199 | 72.7 | 0.80 (0.69–0.93) | 1.12 (0.96–1.32) |
| Coronary artery disease^2^ |  |  |  |  |
| No |  |  |  |  |
| Primary | 17,807 | 101.8 | Ref |  |
| Secondary | 8,209 | 64.7 | 0.89 (0.87–0.92) |  |
| Academic | 2,711 | 41.9 | 0.70 (0.68–0.73) |  |
| Yes |  |  |  |  |
| Primary | 9,531 | 128.2 | Ref | Ref |
| Secondary | 4,211 | 96.9 | 0.88 (0.85–0.92) | 0.99 (0.94–1.04) |
| Academic | 1,138 | 69.5 | 0.74 (0.70–0.79) | 1.05 (0.98–1.13) |
| Heart failure |  |  |  |  |
| No |  |  |  |  |
| Primary | 16,371 | 87.2 | Ref |  |
| Secondary | 7,759 | 56.8 | 0.88 (0.85–0.90) |  |
| Academic | 2,577 | 37.3 | 0.69 (0.66–0.72) |  |
| Yes |  |  |  |  |
| Primary | 10,967 | 178.2 | Ref | Ref |
| Secondary | 4,661 | 138.2 | 0.90 (0.87–0.94) | 1.03 (0.98–1.08) |
| Academic | 1,272 | 106.6 | 0.76 (0.72–0.81) | 1.10 (1.02–1.18) |
| Cerebrovascular event |  |  |  |  |
| No 30 days–2.5 years follow-up |  |  |  |  |
| Primary | 14,166 | 109.7 | Ref |  |
| Secondary | 6,526 | 72.7 | 0.89 (0.86–0.92) |  |
| Academic | 1,989 | 46.5 | 0.71 (0.67–0.74) |  |
| Yes 30 days–2.5 years follow-up |  |  |  |  |
| Primary | 3,150 | 145.5 | Ref | Ref |
| Secondary | 1,361 | 115.6 | 0.90 (0.84–0.96) | 1.00 (0.93–1.08) |
| Academic | 411 | 82.9 | 0.73 (0.66–0.81) | 1.04 (0.92–1.16) |
| No 2.5–5 years follow-up |  |  |  |  |
| Primary | 8,145 | 94.8 | Ref |  |
| Secondary | 3,755 | 60.9 | 0.90 (0.85–0.93) |  |
| Academic | 1,174 | 38.8 | 0.70 (0.66–0.75) |  |
| Yes 2.5–5 years follow-up |  |  |  |  |
| Primary | 1,877 | 149.1 | Ref | Ref |
| Secondary | 778 | 109.2 | 0.84 (0.77–0.92) | 0.94 (0.86–1.04) |
| Academic | 275 | 90.8 | 0.80 (0.70–0.90) | 1.13 (0.98–1.30) |
| Cancer |  |  |  |  |
| No 30 days–1 year follow-up |  |  |  |  |
| Primary | 6,087 | 105.1 | Ref |  |
| Secondary | 2,642 | 67.8 | 0.85 (0.81–0.89) |  |
| Academic | 750 | 40.8 | 0.64 (0.59–0.69) |  |
| Yes 30 days–1 year follow-up |  |  |  |  |
| Primary | 2,848 | 304.5 | Ref | Ref |
| Secondary | 1,476 | 260.5 | 1.02 (0.95–1.09) | 1.20 (1.10–1.30) |
| Academic | 494 | 208.3 | 0.91 (0.83–1.01) | 1.42 (1.26–1.62) |
| No 1–2.5 years follow-up |  |  |  |  |
| Primary | 6,553 | 89.0 | Ref |  |
| Secondary | 2,831 | 55.8 | 0.84 (0.81–0.88) |  |
| Academic | 818 | 33.6 | 0.62 (0.58–0.67) |  |
| Yes 1–2.5 years follow-up |  |  |  |  |
| Primary | 1,828 | 184.9 | Ref | Ref |
| Secondary | 938 | 151.4 | 1.00 (0.92–1.09) | 1.18 (1.08–1.30) |
| Academic | 338 | 127.2 | 0.96 (0.85–1.08) | 1.54 (1.34–1.77) |
| No 2.5–5 years follow-up |  |  |  |  |
| Primary | 8,370 | 94.4 | Ref |  |
| Secondary | 3,734 | 59.8 | 0.87 (0.84–0.91) |  |
| Academic | 1,175 | 38.4 | 0.69 (0.65–0.74) |  |
| Yes 2.5–5 years follow-up |  |  |  |  |
| Primary | 1,652 | 167.6 | Ref | Ref |
| Secondary | 799 | 125.1 | 0.92 (0.84–1.01) | 1.06 (0.96–1.16) |
| Academic | 274 | 101.0 | 0.87 (0.77–0.99) | 1.26 (1.09–1.45) |
| ^1^ Adjusted for age as spline, year of AF diagnosis (1995–99, 2000–04, 2005–08) and AF as main admission diagnosis with stratified Cox regression due to non-proportional hazards and the potential comorbidity variables in the table as well as hypertension, diabetes, peripheral vascular disease, dementia, chronic pulmonary disease, rheumatic disease, mild liver disease, moderate to severe liver disease, hemiplegia/paraplegia, renal disease, HIV, and peptic ulcer. Co-morbidities showing non-proportional hazards was evaluated by follow-up time (30 days–2.5 years, >2.5–5 years or 30 days–1 year,> 1–2.5 years, >2.5–5 years) interactions.  ^2^ Not adjusted for coronary artery disease due to overlap with acute myocardial infarction and vice versa.  HR = hazard ratio; CI = confidence interval; crude rate per 1000 person-years.  Cardiovascular event is a composite of history of ischaemic stroke, transient ischeamic attack, and stroke without further specification. Each of these events were counted only once per patient. | | | | |
